# Sudden Deaths in the Puerperal State, &c.

**Published:** 1857-09

**Authors:** 


### SELECTIONS.

*Sudden Deaths in the Puerperal state, from Dynamic Lesions of the Nervous Centers, etc.* Translated by the Editors, from *L'Union Medicale de la Gironde de Bordeaux.*

[CONCLUDED.]

3d. *Lively Moral Impressions.*—Some observations of sudden death in the puerperal state have been attributed to a lively moral impression. Although the circumstances in which they have occurred have been obscure, we must not pass them by in silence, and we will still see that the nervous system is the chief moving cause. Under the influence of pregnancy, the affective and intellectual faculties of the woman are modified, and present a thousand and one aspects; the least emotion, a lively moral impression, makes its report throughout the whole economy. This modification, which supervenes in the nervous system of the woman who has conceived, is such that it exalts her sensibility and renders her more susceptible to the action of physical and moral causes. The work of Mr. Mac Clintock contains two examples of sudden death in consequence of this lively moral emotion. "In a great number of cases," says he, "in which a state of collapse and prostration shows itself, the patients had presented, sometime formerly, a particular moral state, a kind of fatal presentiment, which contributed to this fatal termination." That a long preoccupation of the mind by a dominant idea, must exercise a marked depressive influence on the vital energy, is a fact known by all physicians, and of which we might cite a great number of examples.

"I am convinced of this fact," says Ramsbotham, that the existence of constant despair during the latter period of the pregnancy has a most marked influence in diminishing the happy effects of those powers, by virtue of which are completed the necessary changes which follow labor."

Travers goes so far as to admit that, among the women recently delivered we observe cases which certainly demonstrate the grievous influence of this depressing cause, and in support of which, he reports two facts, of which I cite only the following:

Observation 12th. A young lady, happily married, impressed, probably, by some sorrowful and unexpected accident supervening in the circle of her friends, had manifested, from the commencement of her pregnancy, a fear of dying in her *accouchement,* and although there was no ground for this belief, her fears increased, so as greatly to alarm her relations and friends. She was delivered by a very careful and experienced physician, who was related to her. The labor was easy and natural in all respects; it was not accompanied by any unfavorable circumstance; the infant was dead-born, incompletely developed. The mother died suddenly, six hours after delivery. The body was examined, and did not present any trace of lesion.—*Union Medicale,* 1853.

In the puerperal state, the imagination of the woman is more excitable, more disposed to be alarmed; her morals and sentiments receive profound and particular modifications.

These disorders of the moral and affective faculties are very pronounced, and they are as much more so as the education, mode of life, and organization have impressed on her a greater excitability.

How many women, who, under the influence of pregnancy, and even after delivery, are a prey to distressing dreams, spasms, sadness, spleen, melancholy, and to a state of moral excitability difficult to calm? It is sufficient, in this state of puerperal susceptibility, to have a light occasional cause, the least emotion of joy or pain, to produce the gravest consequences in a very short time. Such is the case cited by M. Dehous, which was communicated to him by M. Nelaton.

Observation 13th. Fifteen days after a natural *accouchement*, a young lady from pure precaution, continued to keep her bed. Her father came to see her, and talked some time with her; she was very well. The nurse went to the door with the father, and when she returned to the bedside of the lady she found her dead. There was no autopsy.

Here is another fact of the same kind, communicated to me lately by Dr. Charmois, of the department of Bayou:

Observation 14th. On the 15th October, '56, I was called to the house of Capt. M. G. to observe the death of his wife, who had died suddenly. This lady, aged 34 years, had an excellent constitution. Delivered twelve days previously, very happily, the lochia had been abundant. She had not experienced any accident. This lady was of a very great sensibility; to such a degree that one day, from a simple contrariety, while she had her menses, they were arrested and did not return for six months. She received a letter from her husband, who had been absent from the beginning of her pregnancy, in which he announced his arrival on this day. She was greatly excited. At nine A. M., she received him without manifesting very great joy; she conversed all day with him. About seven P. M. was placed in another bed, and suddenly fainted and died. All means usually employed were useless. I arrived two hours afterward to look on a dead body. She was fat, and her face did not show any traces of suffering.

In presence of all these observations, so full of interest, can we not ask, if some of these cases of death, not yet explained, and that we have not wished to relate, for fear of making our paper too long, are not due to one of these lively moral emotions? There are, we know, some obscure facts, whose explanation seems to belong as much to psychology as to physiology, in which the most exercised scalpel, and all the means of investigation of anatomical analysis, have not been able to discover the causes. It would be a vain pretension on the part of the pathological anatomist to try to find constantly a material alteration, palpable in the organs to explain these fatal terminations in the woman who is confined, or who is still under the influence of the puerperal state. The pathological anatomist is forced to recognize his inability there, or perhaps his means of experiment, insufficient and confined, force him to stop. It is permitted to the physiologist to study the mechanism of these moral causes, in the development of certain diseases, and in the production of several deaths. Pain and joy kill, and this truth is not only of the domain of poetry, but it belongs as entirely to that of surgery in general. The moral apparatus of the woman has its education, its disposition, its proper diseases. If the cerebro-spinal mass is identical in all as to its anatomical organization, it must be for its effects, its manifestations, its language; it is modified, on the contrary, under the influence of education, and it explains to us why, in the puerperal state, with some nervous and excitable women, their imagination, more easy to be moved, more susceptible of exaltation, abandons itself more easily to all the excess, perverts itself and goes astray. We find, in the work of M. Foissac, on Meteorology, a brilliant parallel of these kinds of organization. "We see," says he, "certain constitutions drawn from these delicate influences, and, for example, those persons, in quite a large number, who feel and thirst as they digest, whom physical storms no more than moral accidents either trouble nor divert from their accustomed way, and whose life enclosed in the realities of *positiveism,* neither know the digression of imagination, nor the multiform shades of sensibility.

The preceding reflections are applicable to these natures. Shall I say unhappy? Shall I say privileged? For whom the sum of happiness and of suffering is double, from their manner of feeling them; they apply themselves to those sensitive intellectual persons for whom a light thorn, physical or moral, is a sharp harpoon; to those persons, in fine, devoted to study and contemplation, anxious for the past, gloomy for the future, and more or less oppressed by the *tædium vitæ*, which penetrates the heart, as the worm the calix of the flower or the fruit ripened by summer."

As to the etiology of sudden deaths, in consequence of moral emotions during the puerperal state, we do not make a false approach when we compare them to the sudden deaths, the consequence of physical pain and nervous exhaustion. In the case before us, the nervous system, worn out also, no longer sends to the necessary organs for the support of life the influx which is indispensable to maintain in play the wheels of the economy. The causes of death in consequence of lively moral emotions exist, according to Cullen, in the brain. According to Bechat, the moral emotions affect the brain but secondarily; it is the heart which ceases to act the first in these cases, and after it, the death of the other organs; the emotion of joy or pain bears on it specially their influence, and as soon as its movement is arrested, very soon all the parts become immovable.

Whether it may be from a cause in the heart or brain that death takes place, it is none the less true that it is always under the influence of the nervous system, from a dynamic lesion of the cerebro-spinal organ to which man owes the place he occupies, in the animal scale.—*Cincinnati Medical Observer.*

